# Dynamics of *Plasmodium falciparium* and *Plasmodium vivax*in a micro-ecological setting, Southwest Ethiopia: effects of altitude and proximity to a dam

**DOI:** 10.1186/s12879-014-0625-x

**Published:** 2014-11-19

**Authors:** Lelisa Sena, Wakgari Deressa, Ahmed Ali

**Affiliations:** Department of Epidemiology, College of Public Health and Medical Sciences, Jimma University, Jimma, Ethiopia; Department of Preventive Medicine, School of Public Health, College of Health Sciences, Addis Ababa University, Addis Ababa, Ethiopia

**Keywords:** Ethiopia, Gilgel Gibe, Malaria episodes, Spatiotemporal dynamics

## Abstract

**Background:**

Refining the spatial and temporal data on malaria transmissions at a defined ecological setting has practical implications for targeted malaria control and enhancing efficient allocation of resources. Spatial and temporal distribution of *P. falciparium* and *P. vivax* were explored around the Gilgel Gibe Hydroelectric Dam (GGHD) in southwest Ethiopia.

**Methods:**

A review of confirmed malaria episodes recorded over eight years at primary health services was conducted. Using individual identifiers and village names malaria records were cross-linked to location and individual records of Gilgel Gibe Health and Demographic Surveillance System (HDSS) data, which had already been geo-referenced. The study setting was categorized in to buffer zones with distance interval of one kilometer. Similarly, altitude of the area was categorized considering 100 meters height intervals. Incidence rate ratios were estimated using Poisson model for the buffer zones and for the altitudinal levels by adjusting for the underlying population density as an offset variable. Yearly temporal variations of all confirmed malaria cases were also evaluated based on the Poisson model using STATA statistical software version 12.

**Results:**

A considerable proportion (45.0%) of the *P. falciparium* episodes were registered within one kilometer radius of the GGHD. *P. falciparium* showed increment with distance from the GGHD up to five kilometers and with altitude above 1900 meters while *P. vivax* exhibited the increase with distance but, decrease with the altitude. Both species showed significantly higher infection among males than females (P <0.01). Temporally, malaria episodes manifested significant increments in the years between 2006/7 to 2009/10 while reduction of the malaria episodes was indicated during 2004/5, 2005/6 and 2010/11 compared to 2003/4 (P <0.01). On average, *P. vivax* was 52% less than *P. falciparium* over the time period considered. P. vivax was significantly higher in the years 2004/5 to 2007/8 and 2010/11 (P <0.001).

**Conclusions:**

Spatial and temporal variations of malaria were observed. The spatial and temporal variations of malaria episodes were also different for the two main malaria species in the area.

## Background

The level of malaria occurrence is determined by the interactions between *Plasmodium* parasite, the *Anopheles* mosquito vector and the human host. The cycle of interactions, and consequently, the malaria transmission dynamics is determined by the ecological niche even in a small geographical area [1],[2]. Hence, anthropogenic interference of the physical environment inevitably modifies the existing equilibrium cycle of the disease transmission pattern [3]. Since malaria transmission intensity varies geographically, the distribution of cases and subsequent morbidity rate may exhibit systematic spatial variation [4]. Therefore, the need for understanding spatial heterogeneity and temporal variations of malaria at micro- geographic level has been emphasized [5]-[7] for targeted control measure applications.

Currently, Ethiopia is undertaking construction of large hydroelectric dams [8],[9] and irrigation schemes of different scales [10]-[12] as part of developmental activities. This in turn can have impact on vector borne diseases, including malaria as the result of environmental modification. Due to human imposed environmental modifications, risk of malaria has been predicted to increase in East Africa [13],[14]; on the other hand, well designed water projects are taught to economically benefit the residents of the dams vicinity and enable the community to strengthen the disease control programmes to overcome the adverse health effects [15],[16]. However, the extent of health impact of hydroelectric dams on local communities closer to such dams is less studied.

A couple of local studies indicated increased risks on malaria were associated with proximity to water development schemes [17]-[19] all those studies were of cross-sectional nature in design to capture temporal influence. However, a follow up study on children two to nine years reported that risk of malaria infection due to proximity to hydro-power dam was insignificant [20]. Still, a few other longitudinal studies [21],[22] reported presence of malaria hot spots and heterogeneity of the disease at some relatively wider intervals. Altitudinal rise of the disease has been reported by some studies [23],[24], though not reported usually by species. Moreover, studies done elsewhere [25]-[27] reported that proximity to water sources was not found to be risk factor for malaria, but other factors.

Hence, identification of those at risk areas and seasons provides strategies for targeting timely and locally tailored interventions [28],[29]. Quantitative description of the spatiotemporal dynamics has practical implications for the development of malaria control and efficient allocation of resources [30],[31]. Spatial investigation of geographic limits of transmission is also used to develop a predictive spatial model of malaria transmission, to facilitate a malaria control strategy based on geographic stratification [32]. To quantify the contribution made by operational intervention and effect of environmental factors, a clear knowledge about the dynamics of malaria in a particular area is important to plan better prevention and management strategies [33]. Therefore, this study was conducted to examine the role of proximity to a hydroelectric dam and altitude on the spatial distribution of *P. falciparium* and *P. vivax*.

## Methods

### Study setting

This record review was conducted in an area surrounding a man-made lake, Gilgel Gibe Hydroelectric Dam (GGHD), which has been operational since 2004 [34]. The area is a Health and Demographic Surveillance Site (HDSS) [35] which includes surrounding villages within ten km radius of the GGHD with about 50,000 residents used to live in the villages. The area is found within altitude range which favors seasonal malaria transmission [17],[20]. Detailed descriptions of the study setting have also been given elsewhere [17],[36],[37].

### Study design

A retrospective record review study was employed using data from local health services (health centers and health posts). This was done by reviewing malaria morbidity records of local health facilities in villages included into the Gilgel Gibe HDSS. Using individual identifiers (name, age, and sex) and village names, malaria positive cases were cross-linked by careful inspection to location and individual records of the HDSS data which had already been geo-referenced (each house in the HDSS has geographic information system (GPS) records).

### Source of data

In Ethiopia, malaria cases are treated both clinically and as confirmed malaria in accordance with the National Guideline [38],[39], depending on the degree of diagnostic capabilities at different levels of the healthcare system. Both the presumptive and confirmed cases are registered on preformatted registration books (log books) at health care levels and reported both weekly as well as monthly to the next higher level of health management system. The current study included all malaria positive records of the local primary healthcare units that were within the Gilgel Gibe DHSS villages, registered by the primary health care units between September 2003 and August 2011 was reviewed. The timeline consideration was based on data availability and the fact that significant change in the number of malaria cases, such as epidemics, occurs in cyclic fashion from five to eight-year periods in the country [40]-[42], in order that any fluctuation of such cycle could be captured.

### Data collection techniques

A format was prepared on a computer spreadsheet (Excel) to collect the secondary data from log books of local government primary health care units. Individual level data on malaria morbidity; such as, diagnosis results (negative, species of *Plasmodium* for positives); dates of diagnoses; available demographic data (residence, age, sex) were registered on the computer spreadsheet. All records of malaria cases who visited the health institutions during the designated time were included in the study. Records of cases with incomplete records, such as dates of health service visit, age, address, results of diagnosis, were excluded from the analysis. Furthermore, cases whose names, sex and age did not relate to the geo-referenced data of the Gilgel Gibe HDSS were excluded from the spatial analysis. Accordingly, a total of 10,443 records of malaria positive cases were linked to the geo-referenced HDSS data, which has data on household member list with individual, household and location identities (IDs) as well as GPS readings.

### Data management and processing

The data on malaria positive records of the healthcare institutions of the study site were transferred to Excel spreadsheet, checked for completeness (date of diagnosis, test results, patient age and address), coded; then, prepared data of malaria cases were exported to STATA statistical software version 12. Episodes of malaria were considered instead of number of cases for the analysis. The study setting was categorized in to five buffer zones considering distance interval of one kilometer. The altitude of the area was also categorized into six levels as up to 1700, 1701 to 1800, 1801 to 1900, 1901 to 2000, 2001 to 2100, and above 2100 meter above sea level (masl) taking into account the narrow range of this micro-epidemiological setting.

Incidence rate ratio (IRR), which is equivalent to odds ratio in the Poison regression, was estimated using Discrete Poisson model for the buffer zones and for the altitudinal levels. The numbers of base population in the buffer zones and in the ranges of altitude were incorporated as an offset variable to adjust for the underlying population density while computing the IRRs among both the buffer zones and ranges of the altitude. Furthermore, the inter-annual trend of the malaria episodes over the study years considered was analyzed both for all positive cases and for *P. vivax* malaria relative to *P. falciparium* using the IRR to detect change in magnitude of the malaria episodes by years relative to the initial year.

### Ethical considerations

Ethical clearances were obtained from the Addis Ababa University College of Health Sciences Ethical Review Board and Oromia Regional Health Bureau Ethical Clearance Committee. Jimma Zone Health Office and the respective District Health offices of the study areas as well as local administrators were communicated by formal letters written from the School of Public Health of the Addis Ababa University and Oromia Regional Health Bureau. Individual information was kept confidential and the names of individuals were removed from data and identified only by identification numbers and the results were communicated in an aggregated manner.

## Results

A total of 53,624 malaria episodes were recorded in the Gilgel Gibe HDSS site over a period of eight years of which 25,605 (47.7%) were records of clinical malaria and 28,019 (52.3%) were confirmed malaria episodes. Of the positive cases, *P. falciparium, P. vivax and* mixed *species* constituted 60.4%, 33.6% and 6.0% respectively. Only those records managed to be linked with location identification of the Gilgel Gibe HDSS (10,443 episode records) were analyzed for spatial distribution of the diseases (Table 1) while all the confirmed positive malaria records were considered in the temporal analysis.

**Table 1:** **Distribution of malaria cases by sex, distance from GGHD, altitude and** ***Plasmodium species*** **, southwest Ethiopia, September 2003 to August 2011**

Variables	Categories	Diagnosis (species of ***Plasmodium*** ), N (%*)			Total
		P. falciparium	P. vivax	Mixed	
**Distance**	< 1 km	3,250 (45.0)	427 (18.1)	336 (39.3)	4,013 (38.4)
	1 to 2 km	1,890 (26.2)	629 (26.6)	261 (30.5)	2,780 (26.6)
	2 to 3 km	1,180 (16.3)	473 (20.0)	167 (19.5)	1,820 (17.4)
	3 to 4 km	661 (9.2)	395 (16.7)	68 (8.0)	1,124 (10.8)
	4 to 5 km	243 (3.4)	228 (9.7)	21 (2.5)	492 (4.7)
	> 5 km	2 (0.03)	210 (8.9)	2 (0.2)	214 (2.1)
Altitude	≤ 1700 m	18 (0.3)	74 (3.1)	5 (0.6)	97 (0.9)
	1701-1800 m	5,796 (80.2)	1,755 (74.3)	716 (83.7)	8,267 (79.2)
	1801-1900 m	857 (11.9)	348 (14.7)	77 (9.0)	1,282 (12.3)
	1901-2000 m	302 (4.2)	115 (4.9)	37 (4.3)	454 (4.4)
	2001-2100 m	137 (1.9)	35 (1.5)	15 (1.8)	187 (1.8)
	>2100 m	116 (1.6)	35 (1.5)	5 (0.6)	156 (1.5)
Age (year)	< 5	651 (9.0)	258 (10.9)	85 (9.9)	994 (9.5)
	5-9	968 (13.4)	345 (14.6)	114 (13.3)	1,427 (13.7)
	10-14	1,098 (15.2)	370 (15.7)	116 (13.6)	1,584 (15.2)
	15 +	4,509 (62.4)	1,389 (58.8)	540 (63.2)	6,438 (61.7)
Sex	Male	4,032 (55.8)	1,360 (57.6)	450 (52.8)	5,842 (56.0)
	Female	3,194 (44.2)	1,002 (42.4)	404 (57.2)	4,600 (44.1)
**Total**		7,226 (100)	2,362 (100)	855 (100)	10,443 (100)
		69.2	22.6	8.2	100

*Percentage within diagnosis.

The species composition of the geo-referenced malaria episodes were *P. falciparium* (69.2%), *P. vivax* (22.6%) *and mixed species* (8.2). Generally, the contribution of *P. falciparium* was higher near the GGHD and proportion of *P. vivax* exhibited increment with distance from the GGHD. Overall, 38.4% of the episodes were recorded in a distance of less than one kilometer radius of GGHD of which *P. falciparium* and *P. vivax* accounted 81.0% and 10.0% respectively. In the buffer zone one to two kilometer radius of the GGHD, where about a quarter of the malaria episodes were recorded, *P. falciparium* contributed 68.2% while the proportion of *P. vivax* was 22.5%. In the buffer zone from two to three kilometers radius *P. falciparium* and *P. vivax* accounted for 62.7% and 27.8% respectively. The proportions of *P. falciparium* in the buffer zones from three to four and four to five kilometers were 58.9% and 47.6% while that of *P. vivax* were 34.8% and 48.3% respectively. Beyond five kilometers, the proportion of *P. falciparium* showed sharp decline to 7.3% while that of *P. vivax* rose to 91.8%.

Distribution of malaria episodes showed different patterns within species as well among the buffer zones. Unlike to *P. vivax* (18.1%), 46.4% of the *P. falciparium* and 39.4% of mixed species episodes were registered within one kilometer radius of the GGHD.

The proportions of *P. falciparium* and *P. vivax* recorded in the buffer zone range from one to two and two to three kilometers radius of GGHD were similar, which were 26.5% and 14.8% for *P. falciparium* and 26.7% and 20.0% for *P. vivax* respectively. On the other hand, only less than four in ten (3.6%) cases of *P. falciparium* but recognizable proportion (19.5%) of the *P. vivax* episodes were recorded beyond three kilometers radius of the GGHD.

Only less than 0.9% of the malaria episodes were registered to occur in the lower altitude of up to 1700 meter above sea level (masl). Majority (79.2%) of the malaria episodes were registered in the altitude range from 1701-1800 masl where 80.2% of the *P. falciparium* and 74.3% of *P. vivax* malaria episodes were recorded and the composition of *P. falciparium* and *P. vivax* were 70.1% and 21.2% respectively. The next larger number (12.3%) of malaria episodes was registered in the altitude range from 1801-1900 masl. In this altitude range, 11.9% and 14.3% *P. falciparium* and *P. vivax* were recorded and the proportions contributed by *P. falciparium* and *P. vivax* were 66.9% and 27.2% respectively (Table 1).

As distance increases from the GGHD, the occurrence of *P. falciparium* malaria increases significantly up to five kilometers (P <0.001), controlling the effects of altitude, age and gender (Table 2). The *P. falciparium* malaria was 3.8, 5.1, 3.7 and about 3 times more likely to occur in the buffer zones from one to two km, two to three km, three to four km and four to five km respectively compared to the occurrence of *P. falciparium* malaria within one km buffer zone of the GGHD.

**Table 2:** **Effects of proximity to a dam and altitude on** ***Plasmodium falciparium*** **malaria southwest Ethiopia, September 2003 to August 2011**

Variables	Categories	IRR	Std. Err.	P Value	95% C.I.	
					Lower	Upper
Buffer zones relative to GGHD	< 1 km	1				
	1-2 km	3.82	0.07	< 0.001	3.68	3.96
	2-3 km	5.10	0.12	< 0.001	4.88	5.34
	3-4 km	3.73	0.10	< 0.001	3.53	3.93
	4-5 km	2.97	0.13	< 0.001	2.72	3.24
	>5 km	1.04	0.15	0.81	0.78	1.37
Altitude in meters	≤ 1700	1				
	1701-1800	0.03	0.01	< 0.001	0.02	0.04
	1801-1900	0.93	0.17	0.71	0.65	1.33
	1901-2000	1.61	0. 30	0.01	1.12	2.31
	2001-2100	2.51	0.47	< 0.001	1.74	3.63
	2101-2230	2.64	0. 50	< 0.001	1.82	3.82
Age (year)	< 5	1				
	5-9	1.05	0.04	0.19	0.98	1.12
	10-14	1.10	0.04	< 0.01	1.03	1.17
	15 +	1.04	0.03	0.14	0.99	1.10
Sex	Male	1				
	Female	0.96	0.01	0.01	0.93	0.99

Increasing altitude showed negative correlation with the occurrence of *P. falciparium* malaria up to 1900 masl, but it exhibited positive association as altitude increases above 1900 masl and the correlation was significant (P <0.01). Altitudes in the range of 1901-2000 m, 2001-2100 and above 2100 were 1.6, 2.5 and 2.6 times more likely to increase the risk of *P. falciparium* malaria compared to altitude up to 1700 masl. Regarding to the risk of *P. falciparium* infection among different age groups, children in the age range ten to fourteen years were affected more significantly than the other age groups (P = 0.04). The risk of *P. falciparium* malaria was seen to be significantly higher among males than females (P <0.01) when the effects of distance from the GGHD, altitude and age are controlled (Table 2).

On the other hand, as distance from the GGHD increases, the occurrence of *P. vivax* malaria increases consistently and significantly (P <0.001), keeping the effects of altitude and gender constant. The occurrence of malaria in the buffer zones from one to two km, two to three km, three to four km, four to five km and beyond five km was 9.8 times, 13.6 times, 11.9 times, 52.0 times and 68.7 times more likely to occur compared to the occurrence of *P. vivax* malaria in the buffer zone within one km radius of the GGHD. However, increasing altitude was negatively and significantly associated with the occurrence of *P. vivax* malaria (P <0.001).

Children under the age of five years were found to be more affected by *P. vivax* malaria than the other age categories. The risk of *P. vivax* among children five to nine years, ten to fourteen years and adult age groups was less by about 9%, 16% and 11% respectively compared to the risk of *P. vivax* in the under five years of age children. There was also higher chance of *P. vivax* malaria risk among males compared to females keeping the effects of altitude, distance from GGHD and age constant (Table 3).

**Table 3:** **Effects of proximity to a dam and altitude on** ***Plasmodium vivax*** **malaria southwest Ethiopia, September 2003 to August 2011**

Variables	Categories	IRR of ***Pv***	Std. Err.	P Value	95% C.I.	
					Lower	Upper
Buffer zones relative to GGHD	< 1 km	1				
	1-2 km	9.82	0.32	< 0.001	9.22	10.47
	2-3 km	13.56	0.50	< 0.001	12.61	14.58
	3-4 km	11.94	0.49	< 0.001	11.02	12.93
	4-5 km	52.00	2.10	< 0.001	48.03	56.29
	>5 km	68.72	3.46	< 0.001	62.26	75.85
Altitude in meters	≤ 1700 m	1				
	1701-1800	0.02	0.001	< 0.001	0.02	0.01
	1801-1900	0.44	0.03	< 0.001	0.38	0.50
	1901-2000	0.64	0.05	< 0.001	0.55	0.75
	2001-2100	0.51	0.06	< 0.001	0.41	0.64
	>2100 m	0.54	0.06	< 0.001	0.43	0.68
Age (year)	< 5	1				
	5-9	0.91	0.04	0.03	0.83	0.99
	10-14	0.84	0.04	< 0.001	0.78	0.92
	15 +	0.89	0.03	0.001	0.83	0.95
Sex	Male	1				
	Female	1.04	0.02	0.04	1.00	1.09

Overall, there was reduction of about 70% and 60% of malaria episodes in the years 2004/5 and 2005/6 respectively compared to that of 2003/4. However, since 2006/7 increment of malaria episodes was evident up to 2010. In 2006/7 the malaria episode increased about 14.9% as compared to 2003/4. In the following three years, the increment of malaria episodes was 2.3 times, 2.9 times and 4.1 times more likely higher in the years 2008/9, 2009/10 and 2011 as compared to the observed episodes of malaria in the year 2003/4. In the year 2011, the malaria episodes showed reduction of 14% which was statistically insignificant (P = 0.30).

On average, *P. vivax* was 52% lower than *P. falciparium* over the study period considered. The rate of *P. vivax* increments were 130% and 40% in the years 2004/5 and 2005/6 respectively as compared to *P. falciparium* episodes those years. In the years 2006/7, 2007/8 and 2010/2011 there were 60% *P. vivax* rate increments compared to *P. falciparium* during the same time. On the other hand, in the years 2008/9 and 2009/10, there were reductions of *P. vivax* episodes rate by 10% and 16% respectively though the reduction was insignificant in 2008/9 (P = 0.06) (Table 4).

**Table 4:** **Trends of confirmed malaria by year and Species southwest Ethiopia, September 2003 to August 2011**

Plasmodium species	Year	IRR	Std. Err.	P value	(95% C. I.)	
All positive	2003/4	1				
	2004/5	0.30	0.02	< 0.001	0.27	0.34
	2005/6	0.40	0.02	< 0.001	0.36	0.44
	2006/7	1.15	0.04	< 0.001	1.07	1.24
	2007/8	2.26	0.08	< 0.001	2.11	2.41
	2008/9	2.87	0.09	< 0.001	3.00	3.06
	2009/10	4.08	0.13	< 0.001	3.84	4.34
	2010/11	0.96	0.04	0.30	0.89	1.04
P. vivax in reference to P. falciparium	During the whole period	1				
		0.48	0.02	< 0.001	0.43	0.52
Rate ratio	2003/4	1				
P. vivax to	2004/5	2.30	0.20	< 0.001	1.95	2.72
P. falciparium	2005/6	1.41	0.12	< 0.001	1.19	1.67
	2006/7	1.57	0.10	< 0.001	1.39	1.78
	2007/8	1.59	0.09	< 0.001	1.42	1.77
	2008/9	0. 90	0.05	0.06	0.80	1.00
	2009/10	0.84	0.05	< 0.01	0.76	0.94
	2010/11	1.61	0.10	< 0.001	1.42	1.83

## Discussions

This study assessed malaria transmission intensity level spatially within the proximity of GGHD taking into account possible influence of altitude on episodes of *P. falciparium* and *P. vivax* malaria. Temporal trend of all positive episodes of malaria over an eight year period was also examined.

There was variation of species with respect to distance from the GGHD and altitudinal levels. From descriptive analysis, it was apparent that most of *P. falciparium* malaria have occurred nearer to the Dam, within two kilometer radius of GGHD and showed reduction with distance from Dam. This finding is in line with previous local studies [18],[43],[44] though the earlier findings were not reported in terms of *plasmodium species* composition. However, adjusting for the population density revealed the risk of *P. falciparium* infection was higher beyond one km radius of the Dam, especially in the buffer zone two to three km of the Dam.

The spatial cluster of *P. falciparium* in a small geographical area is also compatible with other findings [1],[22],[45],[46]. However, the current finding is in contrast to previous local findings, where the risk difference for *P. falciparium* between children who lived within three kilometer of GGHD and beyond eight kilometers was insignificant [17],[20], implying proximity to water bodies alone does not increase risk of the disease. Again, another study reported that distance to water body was reported not to be significantly associated with prevalence of *P. falciparium* malaria [26].

However, the magnitude of *P. falciparium* malaria exhibited increment with altitude above 1900 masl, implying the up scrolling of malaria to highland areas possibly due to change in climate variables as had already been evidenced elsewhere [14],[24]. The fact that *P. falciparium* prevails in the area mainly for short period of time following heavy rainfall from September to November usually in the form of epidemics [47], might have played a role in increasing the number of cases with altitude, as more susceptible individuals are exposed to the infection for relatively shorter duration due to the wave of epidemics.

On the other hand, *P. vivax* malaria episodes exhibited decrease as altitude increased, especially starting from 1800 masl; however, it showed persistently significant increase as distance increases from GGHD (P <0.001). The decrement of *P. vivax* malaria episodes with increasing altitude was similar to another finding [48]. The proportional increment of *P. vivax* malaria episodes with the distance from the Dam was in agreement with a previous local study which indicated that, unlike *P. falciparium* malaria, more *P. vivax* malaria among distant communities was comparable to those close to a large dam [9]; however, the underlying rationale is not clear and needs further explorations as the *P. vivax* has been given less attention and under studied [49] in spite of the fact that it poses potential severe health consequences [50]-[52].

Pertaining to the trend of the disease, *P. falciparium* malaria significantly increased from 2006/7 to 2009/10 (P <0.001), compared to the 2003/4, but it was lower in the years 2004/5, 2005/6 and 2010/11. Such persistent increment of the episodes of *P. falciparium* could possibly be due to resistance development by malaria vector mosquitoes to insecticides that had been in use for indoor residual spraying (IRS) in the country for about half of a century until vector resistance to DDT was detected in 2007 [53]-[55]. The noticed decline of *P. falciparium* malaria episodes after 2010 could possibly be due to the replacement of old LLINs in 2009, and shifting of insecticide used for IRS in 2010 to deltamethrin or bendiocarb insecticides [53], such coincidence of interventions followed by a sharp drop of malaria episodes, had also been reported by other studies [56]-[61] whereas weakened interventions and development of resistances of the vector to insecticides and the parasite to drugs have been documented to associate with the resurgence of the diseases [62].

The decline of *P. falciparium* malaria episodes between 2004/5 and 2005/6 might also be attributed to the scale up of interventions, including LLITNs [63],[64] following the large scale malaria epidemics of 2003 [65],[66] implying the need for monitoring the major malaria intervention tools such as IRS & LLITNs, the effectiveness.

## Conclusions

There were both temporal and spatial variations of malaria episodes during the study period. The rise of malaria episodes between the years 2008 and 2010 was due to *P. falciparium* where the contribution of *P. vivax* especially during 2009 and 2010 was lower compared to 2003/4. Spatial distribution of the two species was different that though both exhibited increase with distance from the Dam, *P. vivax* episodes increased by many folds constantly as distance increases from the Dam. However, *P. vivax* episodes were limited mainly to relatively lower altitude range. Regarding to the increment of *P. falciparium* with altitude and that of *P. vivax* with distance from reservoir of dams, further exploration is needed.

Given the higher proportion of *P. falciparium* that contributed to about two-third of the malaria episodes of the area, and the fact that still more than two-third of the *P. falciparium* occurred within two kilometers radius of the GGHD, it demands a vigilant monitoring of the disease in the area and sustainable interventions. Density of the base population needs to be considered while conducting spatial analysis of diseases, which is mostly overlooked.

## Authors' contributions

LS participated in the study design, undertook the field study, analyzed data and wrote the manuscript. WD participated in the study design, revision of the manuscript and facilitation of administrative issues. AA participated in the study design, revision of the manuscript and facilitation of administrative issues. All the authors have also read the manuscript and approved it to submit for publication.
